# Chemical Characterization, Leishmanicidal Activity and In Vitro Cytotoxicity of the Essential Oil Extracted from *Pectis brevipedunculata* (Gardner) Sch.Bip. and Its Incorporation into Microemulsion Systems

**DOI:** 10.3390/pharmaceutics16010087

**Published:** 2024-01-09

**Authors:** Auxiliadora Cristina Correa Barata Lopes, Jessyane Rodrigues do Nascimento, Marcos Bispo Pinheiro Camara, Aldilene da Silva Lima, Gláucia Laís Nunes Lopes, Matheus Oliveira do Nascimento, Júlia Karla Albuquerque Melo Xavier, Caroline Martins de Jesus, Cáritas de Jesus Silva Mendonça, André Luis Menezes Carvalho, Lucilene Amorim Silva, Cláudia Quintino da Rocha

**Affiliations:** 1 Programa de Pós-Graduação em Biodiversidade e Biotecnologia da Rede BIONORTE (PPG-BIONORTE), Universidade Federal do Maranhão, São Luís 65080-805, MA, Brazil; auxiliadora.lopes@discente.ufma.br (A.C.C.B.L.); marcos.camara@discente.ufma.br (M.B.P.C.); 2 Programa de Pós-Graduação em Química, Universidade Estadual Paulista Júlio de Mesquita Filho, Araraquara 14800-060, SP, Brazil; jessyane.nascimento@unesp.br; 3 Programa de Pós-Graduação em Química, Universidade Federal do Maranhão, São Luís 65080-805, MA, Brazil; aldilene.silva@ufma.br (A.d.S.L.); julia.xavier@icen.ufpa.br (J.K.A.M.X.); cjm.mendonca@ufma.br (C.d.J.S.M.); 4 Programa de Pós-Graduação em Ciências Farmacêuticas, Universidade Federal do Piauí, Teresina 64049-550, PI, Brazil; glaucialais@ufpi.edu.br (G.L.N.L.); matheusodn2@ufpi.edu.br (M.O.d.N.); aluismenezes@ufpi.edu.br (A.L.M.C.); 5 Programa de Pós-Graduação em Saúde e Tecnologia, Universidade Federal do Maranhão, Imperatriz 65900-410, MA, Brazil; caroline.mj@discente.ufma.br; 6 Programa de Pós-Graduação em Ciências da Saúde, Universidade Federal do Maranhão, São Luís 65080-805, MA, Brazil; lucilene.silva@ufma.br

**Keywords:** Asteraceae, formulation, *L.* (L.) *amazonensis*, monoterpenes, nanotechnology, promastigote

## Abstract

*Pectis brevipedunculata* (Gardner) Sch.Bip., known in Brazil as alecrim do campo, is a small Asteraceae family plant with a calming effect and consumed as tea. This species contains components, such as neral and geranial, that display various biological activities, such as leishmanicidal. The aim was to chemically characterize the essential oil (EO) obtained from *P. brevipedunculata* (EO-PB) by hydrodistillation and a microemulsion formulated with EO (ME-PB), Tween 80 and Transcutol P, assess the leishmanicidal effect against *Leishmania* (L.) *amazonensis* promastigotes and cytotoxicity against RAW 264.7. EO-PB and ME-PB were analyzed by Gas Chromatography Mass Spectrometry (GC/MS). Monoterpene hydrocarbons were noteworthy among the identified compounds. The main EO-PB constituents were α-pinene and limonene, followed by neral and geranial, which were maintained in ME-PB. EO-PB presented an inhibitory concentration (IC_50_) of 20 µg/mL and ME-PB of 0.93 µg/mL. ME-PB inhibition towards the parasite was 20-fold higher than that of EO-PB. This indicated that EO incorporation to the microemulsion resulted in optimized biological activity. Selectivity indices indicate that ME-PB is more selective concerning parasite inhibition. Thus, ME-PB may comprise an adequate approach against Leishmania, as the inhibitory concentration (IC_50_) promastigotes was lower than that considered toxic for cells cell cytotoxicity of 50% (CC_50_).

## 1. Introduction

*Pectis brevipedunculata* (Gardner) Sch.Bip., popularly known in Brazil as alecrim do campo, capim-santo, catinga-de-formiga, limãozinho and capim-limão [1,2,3], belongs to the *Asteraceae* family and *Pectis* L. genus [3]. This plant is distributed throughout some Brazilian regions, namely in the states of Pará, Maranhão, Piauí, Ceará, Pernambuco, Bahia, Goiás, Brasília, Minas Gerais and Rio de Janeiro, and also distributed in other countries, such as Ecuador, Mexico and the United States [3,4].

*Pectis brevipedunculata* is a small plant displaying spontaneous occurrence in lawns and uncultivated fields [5]. One notable feature of this species is its leaf scent, reminiscent of lemongrass, which is known for its citrusy and lemon-like odor and is often used in culinary and medicinal applications. Regarding its chemical composition, the major *P. brevipedunculata* essential oil (EO) components are geranial and neral, which, together, represent about 51% of the plant’s in natura oil. The third most prevalent component is α-pinene, ranging from 17 to 30%, followed by limonene, from 7 to 14% [6,7].

Identified as the main *P. brevipedunculata* component, citral (neral and geranial) is responsible for this plant’s calming effect and is associated with its popular uses [8]. The literature also reports traditional citral uses for other *Pectis* genus species, such as against anxiety, hypertension, intestinal problems, colds, in flavoring, and as a nematicide, larvicide and antimicrobial agent [9,10]. This indicates that *Pectis* species display biotechnological potential and may be employed as a source of new drugs against diseases, such as leishmaniasis, a worldwide public health problem.

Tegumentary leishmaniasis is an infectious, non-contagious disease that is transmitted to humans during the blood meal of female sandflies (*Phlebotomus* and *Lutzomya genera*) infected with *Leishmania* genus protozoa. This disease causes skin and mucous membrane ulcers in humans. Leishmania infection, however, does not confer immunity and affects 1 million people per year worldwide [11,12]. Leishmaniasis treatment comprises the administration of pentavalent antimonial drugs, amphotericin B and its liposomal formulation AmBisome, as well as Miltefosine, Paromomycin, and Pentamidine. But these drugs present certain limitations, such as parasite resistance and cases of toxicity toward human cells [13,14]. Thus, novel and effective methods of controlling the disease are required.

In this regard, natural products, especially terpenes, are examples of secondary metabolites that may be employed to combat leishmania. These compounds can be incorporated into certain formulations, such as microemulsions (ME), to intensify their effects. ME comprise thermodynamically stable isotropic and optically transparent nanometric systems formed by immiscible fluids, presenting both an aqueous phase and an oil phase stabilized by surfactants [15]. These formulations increase the bioavailability of active principles, optimize their biological activity and reduce compound toxicity, contributing to accelerating release speed and improving the clinical efficacy of drugs. In addition, they also increase nutrient, drug and cosmetic stability and optimize food conservation, among other properties [16].

Based on the above, this study aimed to chemically characterize the essential oil (EO-PB) obtained from *P. brevipedunculata* and a formulated microemulsion employing this oil (ME-PB) while also assessing leishmanicidal activity against *L.* (*Leishmania*) *amazonensis* promastigotes and cytotoxicity towards RAW 264.7 macrophages.

## 2. Materials and Methods

### 2.1. Collection of Plant Material

Aerial parts of *P. brevipedunculata* were collected on the campus of Universidade Federal do Maranhão (UFMA), São Luís, MA, Brazil, coordinates: 2°33′20.5″ S/44°18′32.7″ W. A voucher specimen was deposited in the Herbarium Rosa Mochel (SLUI), Universidade Estadual do Maranhão (UEMA), São Luís, MA, Brazil, under the No. 5287. The plant is registered in the National System for the Management of Genetic Heritage and Associated Traditional Knowledge (SisGen) under number AAFB38B.

### 2.2. Essential Oil Extraction

EO-PB was extracted by hydrodistillation using the Clevenger system from 300 g of the plant sample dried for 48 h at room temperature. The oil was extracted for a period of 3 h, after which it was centrifuged and dried with anhydrous sodium sulfate P.A (ISOFAR, RJ, Brazil). Yield was calculated according to [17].

### 2.3. Oil Composition Analysis and Microemulsion

The analysis of OE-PB and ME-PB was performed by Headspace Gas Chromatography Mass Spectrometry using a gas chromatograph (GC-2010) coupled to a mass spectrometer (GCMS-QP2010 Plus) (Shimadzu, Kyoto, Japan). A DB-5MS capillary column (30 m × 0.25 mm × 0.25 μm) with a helium carrier gas flow rate at a linear velocity of 39.5 cm/s (1.0 mL/min) was used. The oven setting was 40–240 °C (10 °C/min). The injector and ion source temperatures were 260 °C and 200 °C, respectively. The following chromatographic conditions were used: sample volume of 50 µL of the EO-PB sample and 500 μL of the ME-PB. Syringe: 2.5 mL—HS. Incubation temperature: 85 °C. Incubation time: 5 min. Shaking speed: 250 rpm. Filling speed: 100 µL/s. Retention indices were calculated for all volatile components using a homologous series of C_8_–C_20_ n-alkanes (Sigma-Aldrich, St. Louis, MO, USA) according to the linear equation of Van Den Dool and Kratz [18]. The components of the EO and ME were identified by comparison of their mass spectra (molecular mass and fragmentation pattern) with data stored in the Adams and NIST libraries [19,20].

### 2.4. Development of Microemulsion and Incorporation of Essential Oil

To develop the microemulsion, surfactants Tween 80 (Polissorbato 80, Êxodo Cientifica, Sâo Paulo, Brazil) and Transcutol P (Diethylene glycol monoethyl ether, Êxodo Cientifica, São Paulo, Brazil) were used. ME-PB was formulated by mixing the surfactants at a 1:1 ratio under magnetic stirring (SolidSteel, Piracicaba, Brazil) for 2 min at room temperature [21]. Next, the oily phase, the essential oil, was titrated to the surfactants in proportions (weight/weight) 1:9; 2:8; 3:7; 4:6; 5:5; 6:4; 7:3; 8:2 and 9:1 under magnetic stirring for 3 min. For each proportion, distilled water was slowly titrated in quantities of 1; 2; 3; 4; 5; 6; 7; 8 and 9, with constant magnetic stirring for 10 min. The phase behavior of the pseudoternary system of the 81 formulations was monitored using a black background for virtual inspection [22]. The stability of the systems was verified after resting for 24 h at room temperature. Then, based on the changes in visual appearance recorded, the pseudoternary phase diagram was constructed, plotting the points in the Origin Pro 8.6 software (OriginLab Corporation, Northampton, EUA). The diagram highlights the regions of the Winsor Classification: WI (Winsor I, two-phase system formed by an oil phase in equilibrium with an emulsified phase); WII (Winsor II, two-phase system formed by an aqueous phase in equilibrium with an emulsified phase); WIII (Winsor III, three-phase system formed by an aqueous phase and an oil phase, intermediated by an emulsified phase); WIV (Winsor IV, single-phase system, the ME region); and a milky white liquid emulsion and cloudy system. From the analysis of the phase diagram, the ideal ME formulation was defined. The formulated ME was stored in a place protected from light, heat and humidity, and after 48 h [23], it was chemically and biologically characterized.

### 2.5. Physicochemical Characterization of the Microemulsion

The droplet size (z-average diameter) and polydispersity index (PDI) were measured by Zetasizer Nano ZS90 (Malvern Instruments, Malvern, UK) at 25 °C at a 90° angle. The observations were performed in triplicate following a proper dilution in a 1:100 proportion (*v*/*v*) in ultra-purified water. The surface charge of the droplets was evaluated by zeta potential in the same instrument [15]. The thermal stability of the ME was analyzed by freezing cycles, in which the sample was subjected to −5 °C ± 2 °C for 24 h in the refrigerator (Electrolux, São Paulo, Brazil), and thawing cycles at 50 °C ± 2 °C for 24 h in an oven (SolidSteel, Piracicaba, Brazil), in a total of 6 cycles for 12 days [24]. The ME was also characterized by density, pH and refractive index, before and after the thermal stability test at 25 °C [15].

### 2.6. Culture of Leishmania (Leishmania) amazonensis N Promastigotes

Schneider’s medium supplemented with 10% Fetal Bovine Serum (FBS) (Gibco, São Paulo, Brazil) was used for parasite culture. Promastigotes of *L.* (L.) *amazonensis* (MHOM/Br/90/BA125) were in the stationary growth phase with flagellar motility. The culture was stored in a Biochemical Oxygen Demand (B.O.D) incubator (Labor, SP-500, São Paulo, Brazil) at 26 °C [25,26].

### 2.7. Leishmanicidal Activity 

Leishmanicida activity was verified by subjecting promastigote forms of *L.* (L.) *amazonensis* (5 × 10^7^ cells/mL) a concentrations varied, such as 8 concentrations for EO-PB (500 to 3.90 μg/mL), 11 for ME-PB (500 to 0.48 μg/mL), and 6 for ME-BLANCK (500 to 15.62 μg/mL), which were added into 96-well plates, containing a final volume of 100 μL. The was carried out in triplicate, according to the methods of [26], with adaptations. The negative control consisted of the promastigote forms grown in Schneider’s medium (Sigma-Aldrich, St. Louis, MO, USA) supplemented with 10% FBS. The positive control was composed of the standard Pentamidine^®^ (Sanofi aventis, Anagni, Italy) (IC_50_: 4 μg/mL) on the parasite grown in the supplemented medium. A control of the medium and a diluent, 0.5% dimethyl sulfoxide (DMSO) (ISOFAR, RJ, Brazil), was also performed with the cultured parasite. The viability of *L.* (L.) *amazonensis* promastigotes and *L. amazonensis* RAW 264.7 macrophages was evaluated by the 3-(4,5-dimethylthiazol-2-yl)-2,5-diphenyltetrazolium bromide tetrazolium (MTT) colorimetric method. This test converts the water-soluble 3-(4,5-dimethyl-2-thiazole)-2,5-diphenyl tetrazolium bromide salt (Sigma-Aldrich, St. Louis, MO, USA), which is yellow in color, into insoluble purple crystals of formazan. Conversion occurs in viable, metabolically active cells through the activity of the mitochondrial enzyme succinate tetrazolium reductase. Thus, it is possible to quantify living cells [27,28]. The 50% inhibitory concentration (IC_50_) was calculated with the absorbance values obtained.

### 2.8. Cytotoxicity Assay on Macrophages

RAW 264.7 macrophage cell lines (2 × 10^6^ cells/mL) cultured at 37 °C and 5% CO_2_ were used in the in vitro cytotoxicity assay, and treated with varied concentrations, such as 8 concentrations for EO-PB (500 to 3.90 μg/mL), 11 for ME-PB (500 to 0.24 μg/mL), and 6 for ME-BLANCK (500 to 15.62 μg/mL) of ME-BLANK in a 96-well plate. The tests were performed in triplicate, according to the methodology of [29], with adaptations. The negative control consisted of cells and an RPMI (Roswell Park Memory Institute) medium (Sigma-Aldrich, São Paulo, Brazil) supplemented with 10% FBS. Positive control was assessed in cells with 0.5% DMSO and the medium used was also controlled. The plates were incubated for 48 h; after this period, the cells were analyzed by the MTT colorimetric method, and read in the microplate reader (BioTek Instruments, Vermont, EUA) at 540 nm. Cell cytotoxicity of 50% (CC_50_) was measured with the absorbance values obtained.

### 2.9. MTT Assay

The MTT method was selected to verify the cell viability of *L.* (L.) *amazonensis* promastigotes and RAW macrophages. In the previous assays, after incubation, the plates were centrifuged, and the supernatant from the wells was removed and replaced with fresh medium containing MTT (5 mg/mL). The plates were then incubated at B.O.D. for 3 h; after this period, the plates were centrifuged again and the formazan crystals were dissolved in 100 μL of pure DMSO. The reading of the absorbances occurred in the microplate reader at 540 nm [30]. To finalize the study, the Selectivity Index (S.I.) was calculated by dividing CC_50_ by IC_50_. A result of S.I. greater than 1 means that the sample shows higher toxicity to the parasite. On the other hand, an S.I. lower than 1 means that the sample has higher toxicity to the cell. Thus, the higher the S.I., the more selective the sample to the parasite and less toxic to the cell [31].

### 2.10. Statistical Analysis

The software used for statistical analysis was GraphPad Prism 8.0 (GraphPad Inc., San Diego, CA, USA). Data were subjected to an analysis of variance (ANOVA) and differences between means were determined by Tukey’s test (*p* ≤ 0.05) in order to obtain the mean, IC_50_ and CC_50_.

## 3. Results and Discussion

### 3.1. Oil and Microemulsion Compositions

The volatile compositions of EO-PB extracted from *P. brevipedunculata* and its ME-PB are displayed in Table 1. Thirty-five compounds were identified by GC-MS, comprising about 97.12% of the total oil composition. The identified chemical compounds are listed in detail, along with their retention times (RT), and Kovats indexes (KI) in Table 1. These EO-PB and ME-PB compounds can be categorized into four classes of components, including high percentages of monoterpene hydrocarbons (83.87%; 84.64%) and oxygenated monoterpenes (9.51%; 8.93%), minor concentrations of fatty acids and derivatives (3.70%, 4.67%) and sesquiterpene hydrocarbons (0.04%; 0.37%).

**Figure 1:**
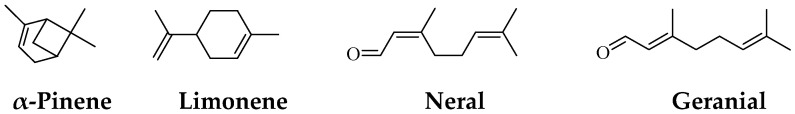
The main constituents of the essential oil (EO-PB) extracted from *Pectis brevipedunculata* and its micro-emulsion (ME-PB) employing the headspace method by Gas Chromatography Coupled to Mass Spectrometry.

The main constituents of EO-PB and ME-PB were α-pinene (56.47%; 50.17%) and limonene (19.98%; 23.17%), followed by citral (7.24%; 8.12%), represented by the sum of two isomeric oxygenated monoterpenes, neral (3.78%; 4.22%) and geranial (3.46%; 3.90%) (Table 1; Figure 1). Citral (neral + geranial) is considered a chemical EO marker for several species belonging to the *Pectis* genus, such as *P. apodocephala, P. angustifolia, P. brevipedunculata, P. ciliares, P. elongata, P. jangadensis* and *P. linifolia* [9].

A recent study on the seasonal and circadian evaluation of the *P. brevipedunculata* EO extracted by hydrodistillation revealed citral (neral and geranial) percentages ranging between 49.8 and 76.4%, followed by α-pinene (7.2% to 19.8%) and limonene (5.3% to 9.4%), both present at lower concentrations [7]. The literature, however, reports that citral concentrations and the volatile profile of *P. brevipedunculata* may vary depending on the EO extraction method [32]. For example, the volatile composition of the *P. brevipedunculata* EO extracted by hydrodistillation contained citral (84.0%), neral (35.8%) and geranial (48.2%), followed by limonene (2.7%) and α-pinene (1.9%), while the EO extracted by headspace-solid phase microextraction method contained high percentages of the monoterpene α-pinene (17.3–25.5%) and limonene (10.0–11.0%), whereas geranial (2.8–10.9%) and neral (2.5–6.5%) were present at only minor concentrations [9].

Citral content variations have also been reported when altering plant drying temperatures. For example, EOs extracted at 40 °C and 60 °C from fresh and dry *P. brevipedunculata* specimens contained neral and geranial as their main constituents, followed by α-pinene and limonene. However, low geranial contents were detected in the EO from plant material dried at room temperature (29.7 °C), with a predominance of α-pinene and neral. This may be due to natural volatilization during long drying times at room temperature, generating EO constituent losses and degradation [6].

### 3.2. Physicochemical Essential Oil Characterization 

EO-PB was characterized concerning yield, density, pH and refractive index. Plant sample moisture contents were also verified after 48 h of collection, immediately before EO-PB extraction. Moisture was determined to be 10.84%, similar to literature reports of about 10% w.b. (wet basis) [6]. In accordance with pharmacopoeias, the recommended maximum plant moisture content is between 8 and 14% [33].

The EO-PB yield extracted from 300 g of aerial *P. brevipedunculata* parts was 0.67%; this percentage was higher compared to specimens reported in other studies, such as aerial parts like leaves, branches, flowers and seeds (0.17%) [34], as well as the EO obtained from leaves and floral portions (0.36%) [6]. A seasonal study of the EO extracted from *P. brevipedunculata* reported higher contents in January and May (2.1%) and lower in February and September (1.1%) [7].

EO-PB density was established to be 0.87 g/mL, in accordance with the established parameters that characterize EOs as ranging from 0.69 to 1.11 g/mL [35]. The refractive index of the investigated EO-PB was 1.44. The EO-PB hydrogen ion potential was 6.00.

### 3.3. Development of Microemulsion and Incorporation of Essential Oil

**Figure 2:**
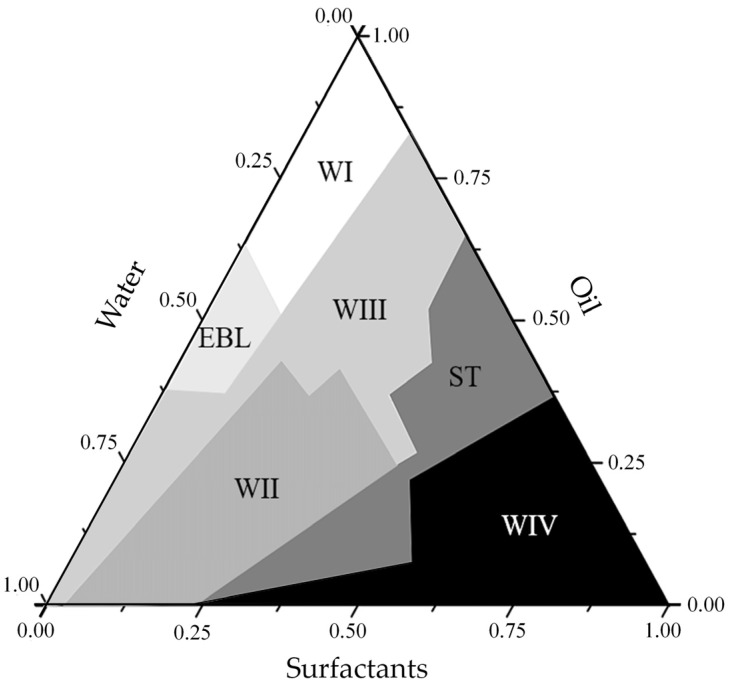
Phase diagram with Winsor Classification (1948): WI (Winsor I, two-phase system formed by an oil phase in equilibrium with an emulsified phase); WII (Winsor II, two-phase system formed by an aqueous phase in equilibrium with an emulsified phase); WIII (Winsor III, three-phase system formed by an aqueous phase and an oil phase, intermediated by an emulsified phase); WIV (Winsor IV, single-phase system, the microemulsion region); EBL (Milky white liquid emulsion); ST (Turbid system).

**Figure 3:**
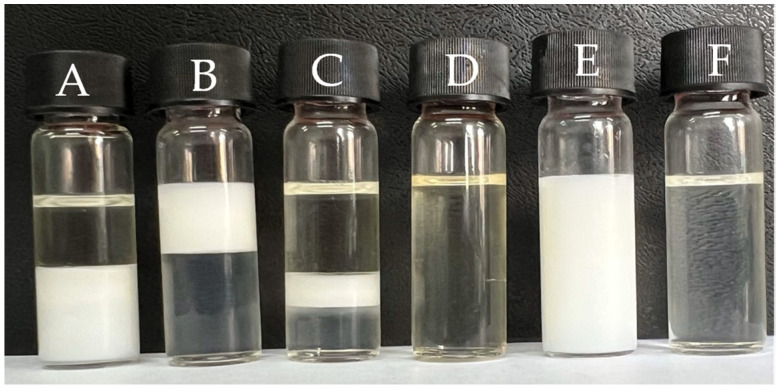
Representation of Winsor Classification (1948). (A) (Winsor I, two-phase system formed by an oil phase in equilibrium with an emulsified phase); (B) (Winsor II, two-phase system formed by an aqueous phase in equilibrium with an emulsified phase); (C) (Winsor III, three-phase system formed by an aqueous phase and an oil phase, intermediated by an emulsified phase); (D) (Winsor IV, single-phase system, the microemulsion region); (E) (Milky white liquid emulsion); (F) (Turbid system).

The systems resulting from the 81 formulations developed with different surfactant: oil: water ratios gave rise to specific regions in the phase diagram (Figure 2), which are the regions of the Winsor Classification: WI (Winsor I, two-phase system formed by an oil phase in equilibrium with an emulsified phase); WII (Winsor II, two-phase system formed by an aqueous phase in equilibrium with an emulsified phase); WIII (Winsor III, three-phase system formed by an aqueous phase and an oil phase, intermediated by an emulsified phase); WIV (Winsor IV, single-phase system); and milky white liquid emulsion and cloudy system (Figure 3).

The single-phase, liquid, optically transparent and stable systems were identified as microemulsions and are allocated in the WIV region of the diagram. From this region of the phase diagram, a total of 18 MEs were obtained, 9 MEs from formulations with a 9:1 ratio, 5 MEs with an 8:2 ratio and 4 MEs with a 7:3 ratio. The selected ME ratio was 7:3:4, which contains the composition presented in Table 2, with the EO incorporated into the system at a concentration of 20%.

### 3.4. Physicochemical Microemulsion Characterization

The microemulsion investigated herein is of the oil/water (O/W) type, consisting of oil droplets dispersed in water and a surfactant at the oil/water interface. This ME category enables compound solubilization and has been employed to achieve higher drug stability, solubility, and bioavailability [36]. ME appearance was visually verified, comprising a transparent, clear, homogeneous system with only one phase, stable up to after 48 h of formulation. After this period, ME-PB was assessed concerning droplet size, polydispersion index, and zeta potential (Table 3).

The nanometric size of ME-PB droplets was 64.75 nm while ME-BLANK droplets were 17.18 nm in size, both in line with what is predicted in the literature concerning particle size, ranging between 10 and 100 nm [37,38,39]. The polydispersion index (PDI) of ME-PB was 0.37 and of ME-BLANK, 0.35, also in accordance with recommended values lower than 0.5, indicating high homogeneity [40]. The verified zeta potentials were −12.9 mV for ME-PB and −25 mV for ME-BLANK. This parameter is associated to particle surface potential and can be negative or positive, depending on the nature of the formulation components (oil and surfactants) [41]. ME-PB was characterized before and after a thermal stability test (Table S1), presenting the same visual characteristics and remaining stable after the test.

A small density variation was verified for ME-PB following heat treatment, decreasing from 0.99 to 0.98 g/mL, while ME-BLANK density increased from 1.00 to 1.02 g/mL. Neither was, however, statistically different and both are by the literature, i.e., microemulsions made with Rosemary, Oregano and Cinnamon EOs exhibited densities of 0.98 to 1.02 g/mL [42].

The pH of ME-PB was 7.09, decreasing significantly to 5.46 following the thermal stability test. The same was noted for ME-BLANK, which decreased from 6.81 to 6.46, albeit non-significantly. Higher aqueous phase contents in oil/water (O/W) ME lead to organic matter ionization, resulting in acidic pH values [15]. The observed pH decrease may be caused by the hydrolysis of system components, such as surfactants and oil phase compounds, or temperature increases [43]. Although parameter variations were noted following the thermal stability test, the pH values were still within the 5.5 to 8.0 range defined as that of greater physicochemical formulation stability [44]. The refractive indices of both ME-PB and ME-BLANK were not altered after the thermal stability test (Table S1).

### 3.5. Leishmanicidal Activity against L. (Leishmania) amazonensis

**Figure 4:**
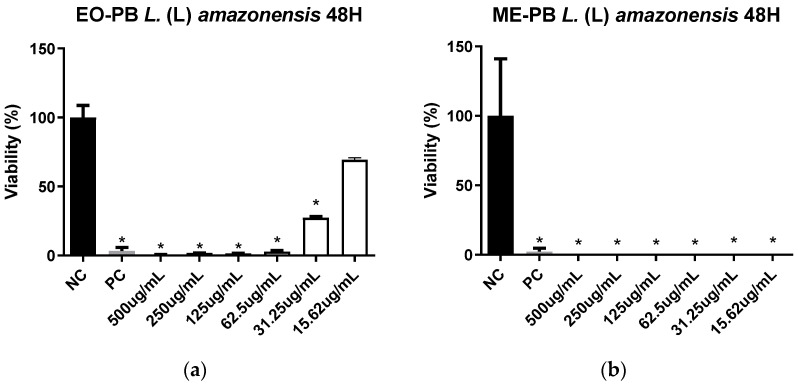
Viability of *L.* (L.) *amazonensis* promastigotes treated for 48 h with essential oil extracted from *P. brevipedunculata* (**a**) and its microemulsion (**b**). NC = negative control; PC = positive control; * Asterisks indicate statistically significant differences about the negative control at *p* < 0.05.

EO-PB displayed leishmanicidal activity against *L*. (L.) *amazonensis* promastigotes, with an IC_50_ of 20 µg/mL, while ME-PB was of 0.93 µg/mL (Figure 4). This indicated about 20-fold higher ME-PB activity compared to EO-PB. ME-PB was also more efficient compared to Pentamidine^®^, a standard drug used as a positive control (IC_50_: 4 µg/mL). On the other hand, ME-BLANK exhibited an IC_50_ of 185.6 μg/mL (Figure S1).

The α-pinene standard, which is one of the major compounds in EO-PB, was also tested and presented an IC_50_ of 64.78 µg/mL. This information was reported in [45], an additional study conducted by our research group. Samples with IC_50_ values equal to or less than 100 μg/mL were defined as active, while samples ranging between 101 and 200 μg/mL were categorized as moderately active, and those over 200 μg/mL were perceived as inactive [46]. Considering this, the EO-PB, the ME-PB and the α-pinene standards investigated herein were configured as active samples, and the ME-BLANK standard was classified as moderately active.

The IC_50_ value of the ME vehicle (ME-BLANK) was higher than that of both EO-PB and ME-PB, indicating that the surfactants employed in the microemulsifying system were not responsible for parasite inhibition and that the observed leishmanicidal ME-PB activity is actually due to active EO principles that become more bioavailable when incorporated into the ME formulation. Active principles effective against *Leishmania amazonensis* as present in *P. brevipedunculata* have also been identified in the EO of several species belonging to different Asteraceae genera. These include *Vanillosmopsis arborea* EO, with an IC_50_ of 7.35 μg/mL, active against promastigotes [47], oils extracted from *Achillea millefolium* (IC_50_: 7.8 μg/mL, leaves and flowers), *Matricaria recutita* (IC_50_: 10.8 μg/mL, flower), *Pluchea carolinensis* (IC_50_: 24.7 μg/mL); *Vernonia brasiliana* (IC_50_: 213 μg/mL, leaves; 112 μg/mL; flowers; 109 μg/mL, root) [48].

Secondary metabolites, such as terpenes, exhibit a mechanism of action that alters organelles, such as the nucleus and mitochondria, interacting and modifying the parasite cell membrane composition and permeability, causing nutrient dysregulation and cell division, potentially leading to cell death [49,50].

### 3.6. Cytotoxicity towards RAW 264.7 Macrophages

**Figure 5:**
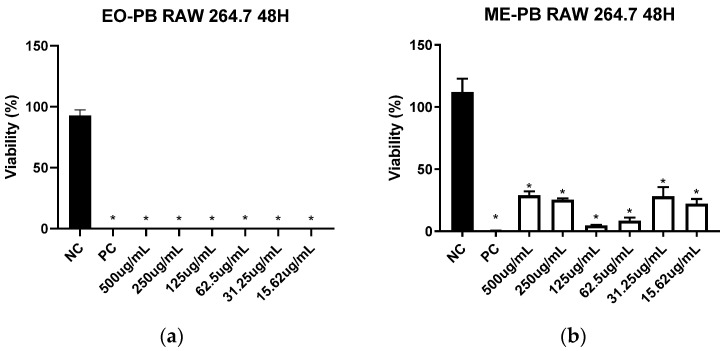
Cytotoxic activity of the essential oil (**a**) and microemulsion (**b**) extracted from *P. brevipedunculata* exerted towards the RAW 264.7 macrophage strain treated for 48 h. NC = negative control; PC = positive control; * Asterisks indicate statistically significant differences in relation to the negative control at *p* < 0.05.

The CC_50_ values of EO-PB, ME-PB and ME-BLANK towards RAW 264.7 macrophages were established as 6.13 µg/mL, 1.33 µg/mL and 62.65 µg/mL, respectively (Figure 5 and Figure S2). Samples with CC_50_ equal to or less than 100 µg/mL were defined as cytotoxic [31]. Thus, the EO-PB, ME-PB and ME-BLANK samples investigated showed potential cytotoxic against the tested cell line.

The Selectivity Index (S.I) was determined based on the cytotoxicity results towards RAW 264.7 cells and IC_50_ against *L*. (L.) *amazonensis*. This index represents the highest sample toxicity or selectivity against parasites or cell lineages [31].

The value of S.I. from EO-PB showed a value lower than 1 (0.30) and the IC_50_ for *L*. (L.) *amazonensis* was higher than that of the CC_50_ (Table 4). This indicates that the investigated EO-PB is more toxic for the investigated cell line compared to the parasite, demonstrating cell disruption selectivity. On the other hand, the ME-PB S.I was higher than 1 (1.43), demonstrating that the assessed ME is more selective for parasite inhibition and less toxic to cells. This confirms the use of formulated ME against *Leishmania*, as the IC_50_ for this protozoan was lower than the CC_50_ (Table 4). ME-BLANK also exhibited an S.I of less than 1 (0.34), similar to EO-PB, resulting in higher cell toxicity against *L*. (L.) *amazonensis*.

The observed toxicities of both EO-PB and ME-PB for cells investigated were, in fact, predicted. The volatile composition of EOs and the surfactants used in ME formulations can alter microorganism cytoplasmic membrane structures and compositions, modifying their permeability and potentially lead to cell rupture [51]. Based on the results explained, OE-PB, when incorporated into a ME system, is safe for use against protozoan Leishmania, as it potentializes the toxicity against the parasite, requiring a lower concentration of ME to inhibit the same amount that would be inhibited by the action of pure OE. This result is obtained because the ME enhances the effects of active ingredients, optimizing their leishmanicidal biological activity.

## 4. Conclusions

The action of active compounds extracted from *P. brevipedunculata* is significantly enhanced in the microemulsion system developed in this study. This system demonstrates a remarkable 20-fold increase in cytotoxicity against *L.* (L.) *amazonensis* promastigotes compared to the essential oil (EO) itself. This innovation may offer a viable substitute for existing drugs used in the treatment of leishmaniasis, which have their limitations, including toxicity to human cells and the development of parasite resistance. In this way, the developed microemulsion represents a significant advancement in harnessing the therapeutic potential of natural plant products for drug development.

## Figures and Tables

**Table 1:** Characterization of the essential oil (EO-PB) extracted from *Pectis brevipedunculata* and its microemulsion (ME-PB) employing the headspace method by Gas Chromatography Coupled to Mass Spectrometry.

					EO-PB	ME-PB
No.	RT ^a^	KI ^b^	Compound	Formula	Area (%)	
1	6.685	855	(*E*)-2-hexenal	C_6_H_10_O	0.05	
2	6.740	853	(3*Z*)-hexenol	C_6_H_12_O	0.08	
3	8.270	926	tricyclene	C_10_H_16_	0.11	0.12
4	8.385	930	α-thujene	C_10_H_16_		0.86
5	8.605	939	α-pinene	C_10_H_16_	56.47	50.17
6	8.895	959	camphene	C_10_H_16_	0.59	0.67
7	9.355	975	sabinene	C_10_H_16_	3.10	3.74
8	9.460	979	β-pinene	C_10_H_16_	1.50	1.87
9	9.595	985	6-methyl-5-heptene-2-one	C_8_H_14_O	3.47	2.47
10	9.680	990	β-myrcene	C_10_H_16_	1.28	2.05
11	9.755	991	6-methyl-5-hepten-2-ol	C_8_H_16_O	0.10	0.05
12	10.375	1024	*p*-cymene	C_10_H_14_	0.12	
13	10.520	1029	limonene	C_10_H_16_	19.98	23.17
14	10.555	1037	(*Z*)-β-ocimene	C_10_H_16_	0.07	
15	10.760	1050	(*E*)-β-ocimene	C_10_H_16_	0.65	1.84
16	10.985	1059	γ-terpinene	C_10_H_16_		0.10
17	11.215	1072	*cis*-linalool oxide (furanoid)	C_10_H_18_O_2_	0.08	
18	11.470	1088	*p*-mentha-2,4(8)-diene	C_10_H_16_		0.08
19	11.550	1370	octylcyclopropane	C_11_H_22_		1.94
20	11.705	1140	*cis*-β-terpineol	C_10_H_18_O	0.26	0.19
21	11.740	1159	α-pinene oxide	C_10_H_16_O	0.63	
22	12.425	1144	*exo*-isocitral	C_10_H_16_O	0.04	0.07
23	12.295	1136	*cis*-limonene oxide (Me vs. IPP)	C_10_H_16_O	0.13	
24	12.360	1142	*trans*-limonene oxide	C_10_H_16_O	0.06	
25	12.715	1144	*trans*-verbenol	C_10_H_16_O	0.16	
26	13.015	1144	(*E*)-isocitral	C_10_H_16_O	0.22	
27	13.720	1229	nerol	C_10_H_18_O	0.22	0.18
28	13.950	1238	neral	C_10_H_16_O	3.78	4.22
29	14.090	1252	geraniol	C_10_H_18_O	0.47	0.29
30	14.385	1267	geranial	C_10_H_16_O	3.46	3.90
31	14.695	1370	*n*-undecanol	C_11_H_24_O		0.21
32	15.920	1375	α-ylangene	C_15_H_24_		0.05
33	16.140	1390	β-elemene	C_15_H_24_	0.04	0.11
34	16.610	1408	(*E*)-caryophyllene	C_15_H_24_		0.12
35	17.085	1454	α-humulene	C_15_H_24_		0.09
			Monoterpene Hydrocarbons		83.87	84.64
			Oxygenated Monoterpenes		9.51	8.93
			Sesquiterpene Hydrocarbons		0.04	0.37
			Fatty acids and Derivatives		3.70	4.67
			Total Identified		97.12	98.61

^a^ RT: Retention Times, ^b^ KI: Kovats retention index.

**Table 2:** Composition of the microemulsion formulated with *Pectis brevipedunculata* essential oil.

Component	Function	Proportion
Tween 80	Surfactant	26.7%
Transcutol P	Co-surfactant	26.7%
EO-PB	Oil phase	20.0%
Distilled water	Aqueous phase	26.6%

**Table 3:** Characterization of the microemulsion formulated using the essential oil extracted from *Pectis brevipedunculata* (ME-PB).

Formulation	Droplet Size (nm)	Polydispersion Index	Zeta Potential (mV)
ME-PB	64.75 ± 22.24	0.37 ± 0.18	−12.9
ME-BLANK	17.18 ± 8.43	0.35 ± 0.07	−25

**Table 4:** Inhibitory concentration (IC_50_), cytotoxic concentrations (CC_50_) and Selectivity Index (S.I.) values of the essential oil extracted from *Pectis brevipedunculata* (EO-PB), its microemulsion (ME-PB) and the vehicle (ME-BLANK) against *L.* (L.) *amazonensis* promastigotes and the 264.7 RAW macrophage strain.

Samples	Promastigote IC_50_ (µg/mL)	Macrophages RAW 264.7 CC_50_ (µg/mL)	S.I.
EO-PB	20	6.13	0.30
ME-PB	0.93	1.33	1.43
ME-BLANK	185.6	62.65	0.34

## Data Availability

Data are contained within the article and Supplementary Materials.

## Supplementary Materials

The following supporting information can be downloaded at: https://www.mdpi.com/article/10.3390/pharmaceutics16010087/s1, Table S1. Characterization of the microemulsion formulated using the essential oil extracted from *Pectis brevipedunculata* (ME-PB) before and after thermal stability test; Figure S1. Viability of *L.* (L.) *amazonensis* promastigotes treated for 48 h the microemulsion blank. NC = negative control; PC = positive control; * Asterisks indicate statistically significant differences in relation to the negative control at *p* < 0.05; Figure S2. Cytotoxic activity of the microemulsion blank exerted towards the RAW 264.7 macrophage strain treated for 48 h. NC = negative control; PC = positive control; * Asterisks indicate statistically significant differences in relation to the negative control at *p* < 0.05.
