# Supplementary material for: Chemical Characterization, Leishmanicidal Activity and In Vitro Cytotoxicity of the Essential Oil Extracted from Pectis brevipedunculata (Gardner) Sch.Bip. and Its Incorporation into Microemulsion Systems
Source: Pharmaceutics. 2024 Jan 9;16(1):87. doi: 10.3390/pharmaceutics16010087 (PMC10818420; doi:10.3390/pharmaceutics16010087)
Supplement: Supplementary file 1 [file pharmaceutics-16-00087-s001.zip › pharmaceutics-2750774-supplementary.pdf]

Supplementary material

Table S1. Characterization of the microemulsion formulated using the essential oil extracted from *Pectis brevipedunculata* (ME-PB) before and after a thermal stability test.

Samples	Density (g/mL)		pH		Refractive Index	
	Before	After	Before	After	Before	After
**ME-PB**	0.99 ± 0[a]	0.98 ± 0.01[a]	7.09 ± 0.23[a]	5.46 ± 0.20[b]	1.43 ± 0[a]	1.43 ± 0[a]
**ME-BLANK**	1.00 ± 0[a]	1.02± 0.02[a]	6.81 ± 0.27[a]	6.46 ± 0.30[a]	1.42 ± 0[a]	1.42 ± 0[a]

The statistical difference between the means of each sample was determined by the Anova two-way test with Tukey's comparison ($p \leq 0.05$), verifying parameters before and after the thermal stability test.

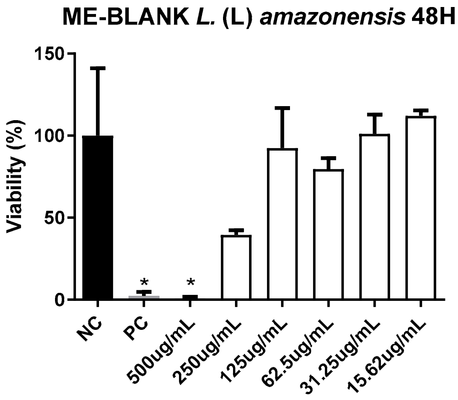

Figure S1. Viability of *L. (L.) amazonensis* promastigotes treated for 48 hours the microemulsion blank. NC = negative control; PC= positive control; *Asterisks indicate statistically significant differences in relation to the negative control at $p < 0.05$.

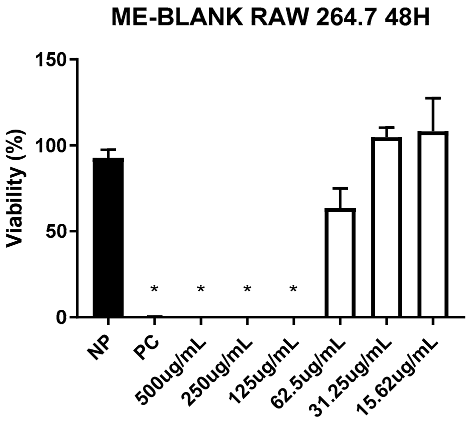

Figure S2. Cytotoxic activity of the microemulsion blank exerted towards the RAW 264.7 macrophage strain treated for 48 hours. NC = negative control; PC= positive control; *Asterisks indicate statistically significant differences in relation to the negative control at $p < 0.05$.
